# Supplementary material for: Firing of Replication Origins Is Disturbed by a CDK4/6 Inhibitor in a pRb-Independent Manner
Source: Int J Mol Sci. 2023 Jun 25;24(13):10629. doi: 10.3390/ijms241310629 (PMC10341899; doi:10.3390/ijms241310629)
Supplement: Supplementary file 1 [file ijms-24-10629-s001.zip › ijms-2456481-proofedSupplementary.pdf]

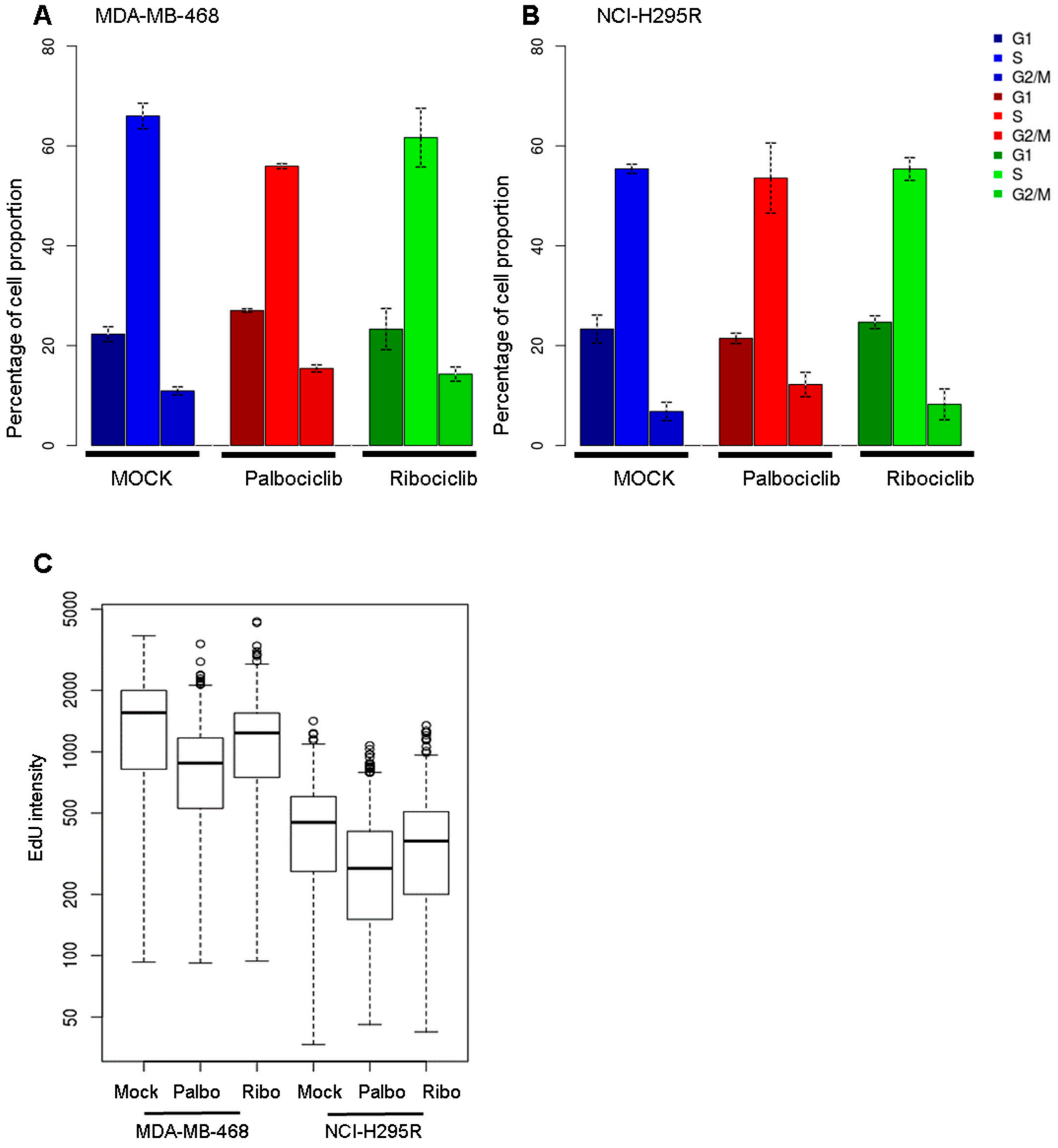

Figure S1.

A. The proportion of each phase of the cell cycle is represented by percentage in MDA-MB-468 cells and **B**. in NCI-H295R cells **C.** Boxplot representing the level of EdU incorporation in S phase in both cell lines. (Palbo=palbociclib treatment, Ribo=ribociclib treatment)

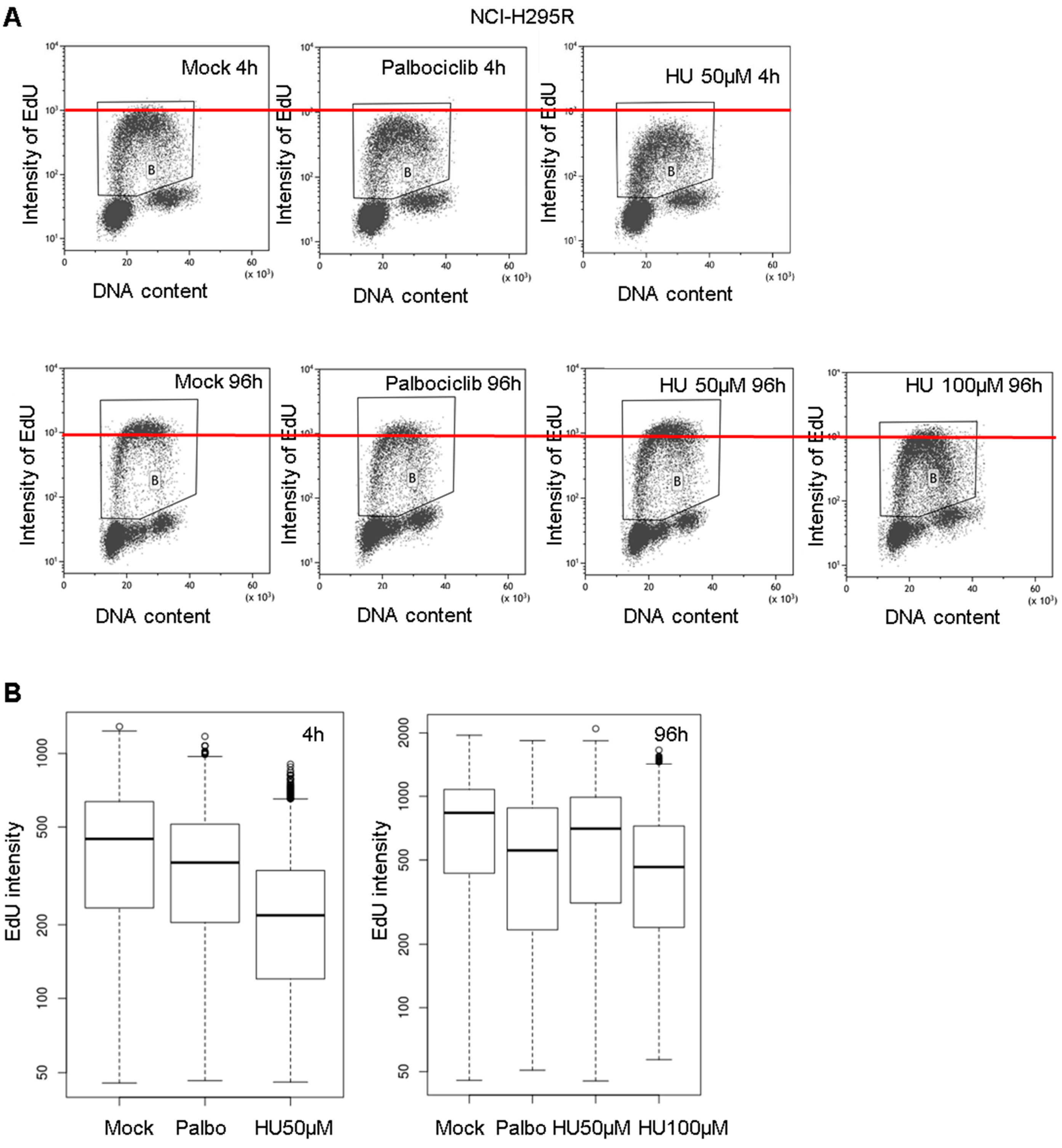

Figure S2.
A. Cell cycle profiles of NCI-H295R cells after EdU incorporation and mock, palbociclib or hydroxyurea treatment for 4 or 96 hours. **B.** Boxplots representing the intensity of EdU incorporation in NCI-H295R cells in S-phase for 4 or 96 hours of treatment. (Palbo=palbociclib treatment, Ribo=ribociclib treatment, HU=Hydroxyurea)

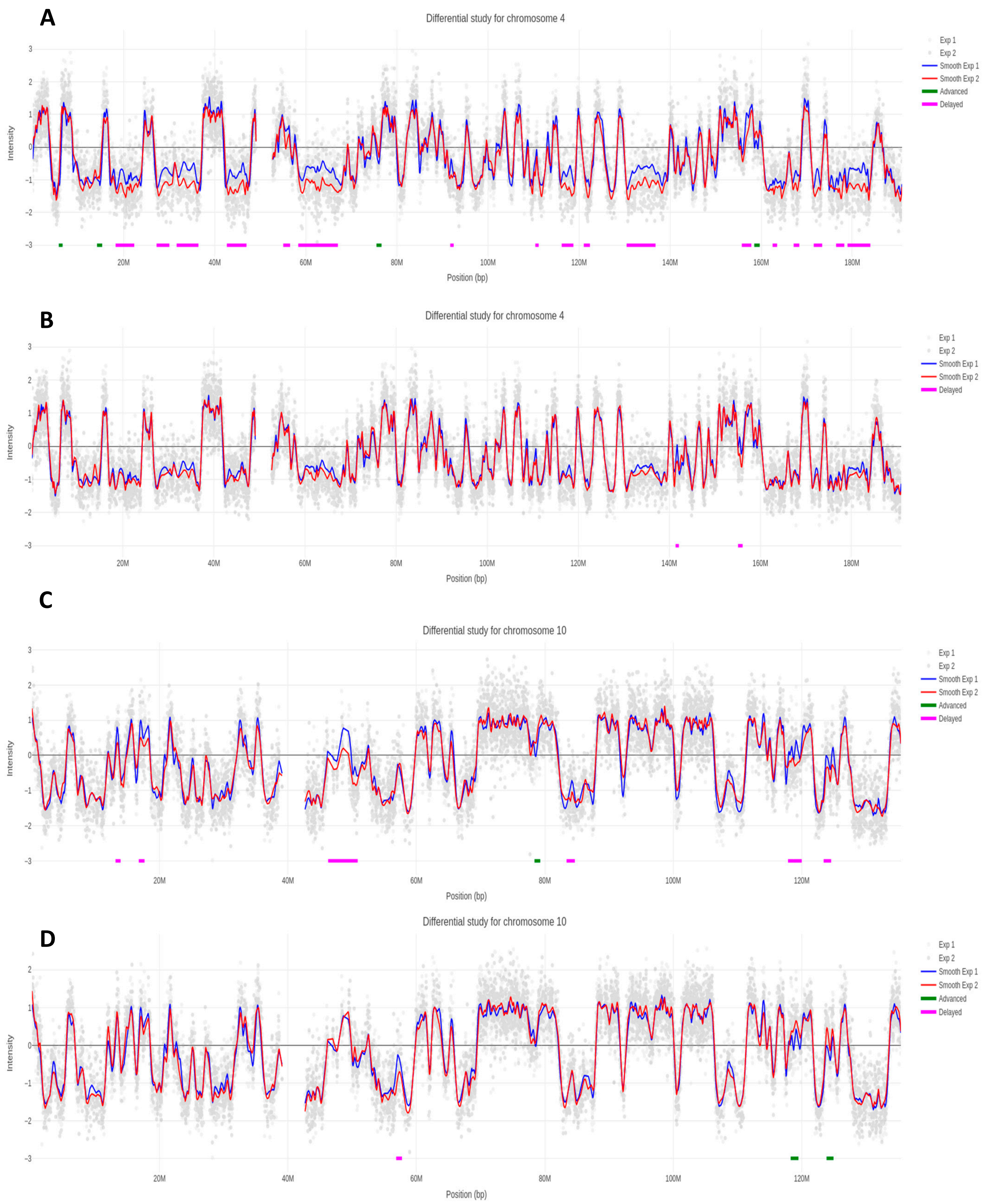

Figure S3. Analysis of the temporal program of DNA replication in MDA-MB-468 and NCI-H295R cells treated with either palbociclib or ribociclib. A and B. Chromosome 4 replication timing profiles in MDA-MB-468 cells. The blue line represents control cells treated with mock, the red one cells treated with palbociclib in A and with ribociclib in B. Chromosome coordinates are indicated below each profile. **C and D.** Chromosome 10 replication timing profiles in NCI-H295R cells. The blue line represents control cells treated with mock, the red one cells treated with palbociclib in A and with ribociclib in B.

A

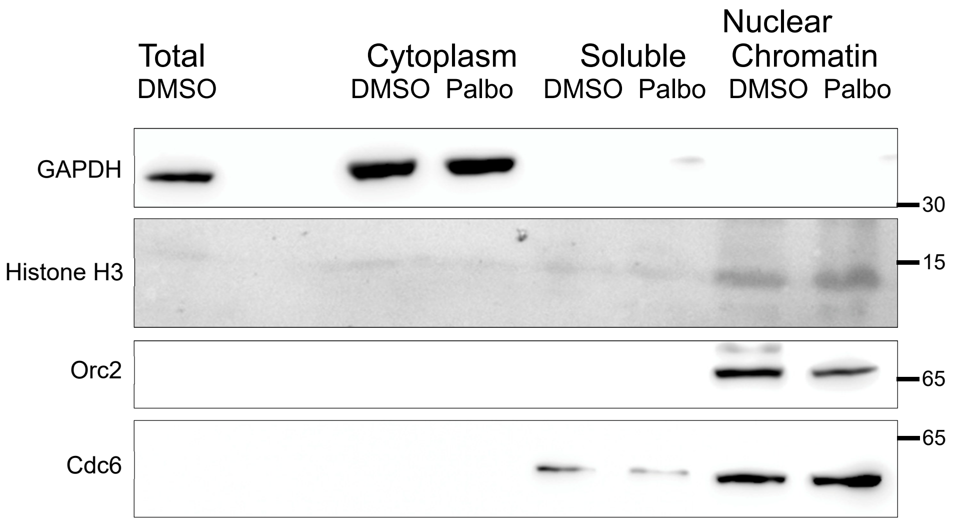

Figure S4.
A. The fractions of proteins were analyzed by Western blotting. The same number of cells are used for fractionation for each condition. DMSO=96 hours of treatment with DMSO and Palbo= 96 hours of treatment in NCI-H295R cells. GAPDH and Histone H3 are used for the control of fractionation.
